# Anticancer activity and metabolite profiling data of *Penicillium janthinellum* KTMT5

**DOI:** 10.1016/j.dib.2019.104959

**Published:** 2019-12-07

**Authors:** Kudzanai Ian Tapfuma, Tendani Edith Sebola, Nkemdinma Uche-Okereafor, Jody Koopman, Raeesa Hussan, Maya Mellisa Makatini, Lukhanyo Mekuto, Vuyo Mavumengwana

**Affiliations:** a DST/NRF Centre of Excellence for Biomedical Tuberculosis Research and SAMRC Centre for Tuberculosis Research, Division of Molecular Biology and Human Genetics, Department of Biomedical Sciences, Stellenbosch University, PO Box 19063, Tygerberg, 7505, Cape Town, South Africa; b Department of Biotechnology and Food Technology, Faculty of Science, University of Johannesburg, PO Box 17011, Doornfontein, Johannesburg, 2028, South Africa; c Molecular Sciences Institute, School of Chemistry, University of the Witwatersrand, PO Box Wits, 2050, Johannesburg, South Africa; d Department of Chemical Engineering, Faculty of Engineering and the Built Environment, University of Johannesburg, PO Box 17011, Doornfontein, Johannesburg, 2028, South Africa

**Keywords:** Mine tailings, *Penicillium janthinellum*, Anticancer, Metabolite profiling, LC-QTOF-MS/MS

## Abstract

Fungi are ubiquitous, they proliferate even in environments with toxic pollutants that are otherwise harmful to other eukaryotes. This article presents data of fungi which were isolated from gold mine tailings and identified by DNA sequencing of their inter transcribed spacer regions 1 and 2. Five fungal isolates were identified, among which the crude extract of *Penicillium janthinellum* KTMT5 was investigated for anticancer activity on A549 (lung carcinoma) and UMG87 (glioblastoma) cell lines. Untargeted metabolite profiling of the crude extract of *P. janthinellum* KTMT5 was performed using liquid chromatography quadrupole time of flight tandem mass spectrometry (LC-QTOF-MS/MS) and a molecular network generated using the online workflow on the Global Natural Product Social molecular networking (GNPS) website. DNA sequencing showed that all fungal isolates belonged to phylum Ascomycota with the genus Penicillium representing 75% of the fungal isolates. *P. janthinellum* KTMT5 which was selected for further experiments showed significant anticancer activity against UMG87 cells with a calculated IC_50_ value of 44.23 μg/mL in the MTS assay, while the real time xCELLigence assay showed dose-dependent anticancer activity at 50 and 100 μg/mL. Metabolite profiling revealed the presence of several known metabolites in the crude extract of *P. janthinellum* KTMT5 and molecular networking showed the relationships among these metabolites.

**Table:** Specifications Table

Subject	Biochemistry
Specific subject area	Metabolomics, Natural Products Research, Spectrometry
Type of data	Tables and figures.
How data were acquired	Anticancer data was acquired using a gold-microelectrode precoated 96 well electronic plate reader (ACEA Biosciences Inc., San Diego, CA, USA). Metabolite profiling data was acquired using a Dionex UltiMate 3000 ultra-high-performance liquid chromatography (Thermo Scientific, Germany) connected to a QTOF (Compact™, Bruker Daltonics, Germany).
Data format	Raw and analysed data.
Parameters for data collection	Conditions considered for data collection were as follows: Potato dextrose medium was incubated at 25 °C, 14 days for agar plates and 21 days for broth shaking at 150 rpm; A549 and UMG87 cancer cells were incubated at 37 °C with 5% CO_2_ (v/v); anticancer activity of *P. janthinellum* KTMT5 was tested at six logarithmic concentrations starting from 3.13 to 100 μg/mL; cancer cells were incubated for 96 hours in the MTS and 250 hours in the xCELLigence assays without media or fungal extract supplementation; the crude extract of *P. janthinellum* KTMT5 was dissolved in methanol at 1 mg/mL for LC-QTOF-MS/MS analysis where the mobile phase consisted of solvent A with 0.1% formic acid in H_2_O (v/v) and solvent B with 0.1% formic acid in acetonitrile (v/v).
Description of data collection	Tailings were collected from a dump of an old and inactive gold mine. The collection was done by drilling 30 cm deep using a surface sterilized auger. Tailings at this depth were used in the isolation of fungi using potato dextrose agar (PDA) and the isolates were identified by sequencing of the inter transcribed spacer regions 1 and 2 (ITS1 and ITS2). These sequences were then blasted on the National Center for Biotechnology Information's (NCBI) GenBank database to get closely matching relatives. The data from the colorimetric end-point MTS assay and the real time xCELLigence assay was analysed using GraphPad Prism software (v. 7.05, GraphPad Software, Inc., La Jolla, CA, USA) while the graphs were plotted using Microsoft Excel (v. 2010, Microsoft Corporation, Washington, USA). Metabolite profiling was achieved by using MetFrag 2.1^1^ while the molecular network was generated on the GNPS online platform.
Data source location	Institution: Stellenbosch University City: Cape Town Country: South Africa Latitude and longitude (and GPS coordinates) for collected samples: -26.218633, 28.485733 (26°13′07.1″S, 28°29′08.6″E).
Data accessibility	With the article.

**Table:** 

**Value of the Data** • The data provides the anticancer activity profiles of the ethyl acetate crude extract of *P. janthinellum* KTMT5 on A549 (lung carcinoma) cells and UMG87 (glioblastoma) human cancer cell lines and the metabolite profile acquired using LC-QTOF-MS/MS. • The data is beneficial to natural product researchers in human cancer drug discovery as it provides the anticancer activity of *P. janthinellum* KTMT5 and the identity of compounds in its extract. • The data in this article may lead to the discovery of compounds with novel mechanisms of action on UMG87 cells, leading to the development of a new drug for glioblastoma multiforme. • In addition to the anticancer and metabolite profile data for *P. janthinellum* KTMT5, this data article provides a reproducible experimental guideline for bioprospecting fungi from mine tailings of different minerals.

## 1 Data description

This data article presents fungi isolated from gold mine tailings of an old and inactive gold mine in Springs, Johannesburg, South Africa (26°13′7.08″S, 28°29′8.64″E). These isolates were identified by sequencing of TS1 and ITS2 regions and matching them with annotated sequences in the GenBank database. Table 1 shows the identity and GenBank accession numbers of these isolates while Supplementary File 1 contains the raw data of the ITS sequences. All species belonged to phylum Ascomycota. The genus Penicillium was the predominant and represented 75% of all the isolates.

**Table 1:** Identities and accession numbers of fungal isolates.

Isolate	GenBank Accession No.	Closest Relative in NCBI	Query Cover (%)	ITS Sequence Similarity (%)	Assigned Identity
KTMT1	MH660411	*Arcopilus aureus* (MG889930)	100	100	*A. aureus* KTMT1
KTMT2	MH660412	*Penicillium janthinellum* (KU529846)	100	100	*P. janthinellum* KTMT2
KTMT4	MH660413	*P. oxalicum (*MK163534)	100	97.94	*P. oxalicum* KTMT4
KTMT5	MH660414	*P. janthinellum* (KM023324)	100	98.51	*P. janthinellum* KTMT5
KTMT6	MH660415	*Acidiella americana* (LT627242)	93	99.02	*A. americana* KTMT6

**Fig. 1:**
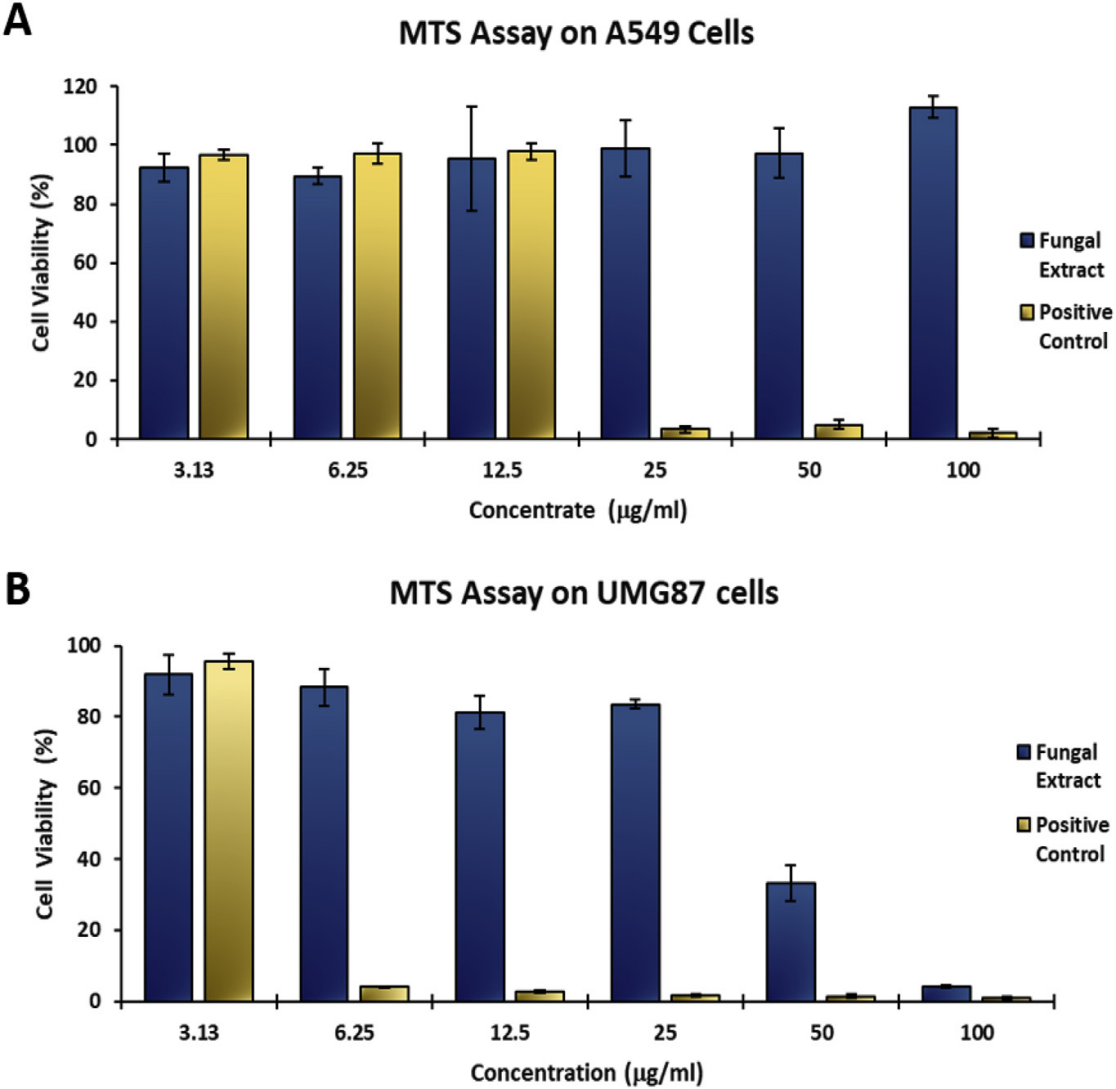
*In vitro* anticancer activity of *P. janthinellum* KTMT5 extract on A549 cells **(A)** and UMG87 cells **(B)**. Error bars in this figure represent the standard deviation (SD) of the mean. The positive control used was auranofin.

*P. janthinellum* isolate KTMT5 was selected for further investigations. Fig. 1 shows the data for *in vitro* anticancer activity screening of the crude extract of *P. janthinellum* KTMT5 performed using the colorimetric MTS (3-[4,5,dimethylthiazol-2-yl]-5-[3-carboxymethoxy-phenyl]-2-[4-sulfophenyl]-2*H*-tetrazolium, inner salt) assay on two human cancer cell lines, namely A549 (lung carcinoma) cells and UMG87 (glioblastoma) cells. A significant reduction in cell viability (cell viability of 4.21 ± 0.26%) for cells treated with *P. janthinellum* KTMT5 crude extract in UMG87 cells was observed at 100 μg/mL. On A549 cells, no observable anti-proliferative bioactivity was observed. The highest concentration treatment of 100 μg/mL on A549 cells was observed to increase cellular metabolism as indicated by a cell viability 113 ± 3.65%. Raw data for the MTS assay can be found in Supplementary File 2.

**Fig. 2:**
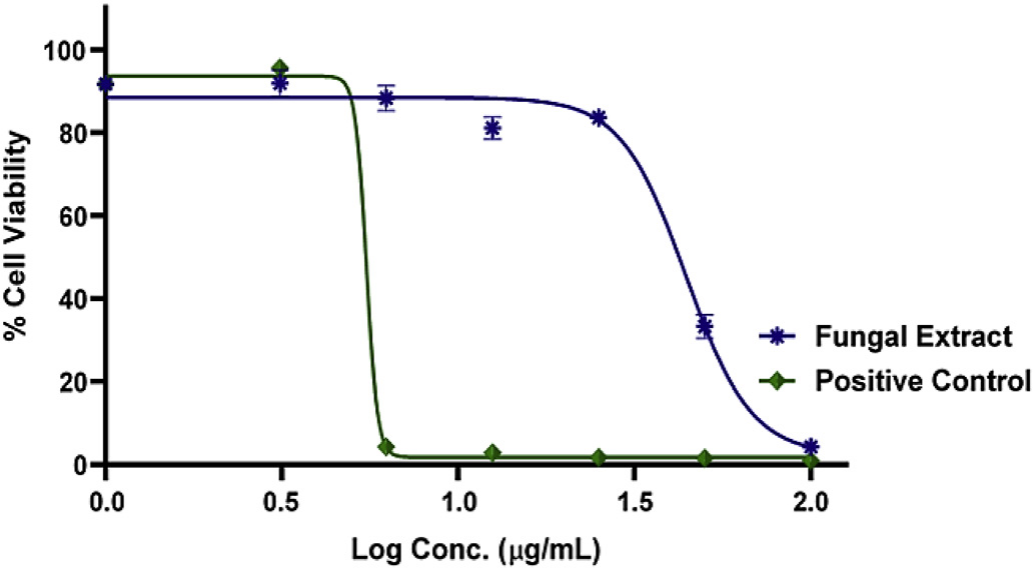
A dose-response curve of the crude extract of *P. janthinellum* KTMT5 (fungal extract) on UMG87 cells. The positive control used was auranofin.

Fig. 2 shows the dose-response curve plotted for UMG87 cells to visualize the effect of the fungal crude extract on this cell line at different logarithmic concentrations. The calculated median inhibitory concentration (IC_50_) value for fungal extract was 44.23 μg/mL and for the positive control auranofin was 5.93 μg/mL. The dose-response curve was plotted using raw data in Supplementary File 2.

**Fig. 3:**
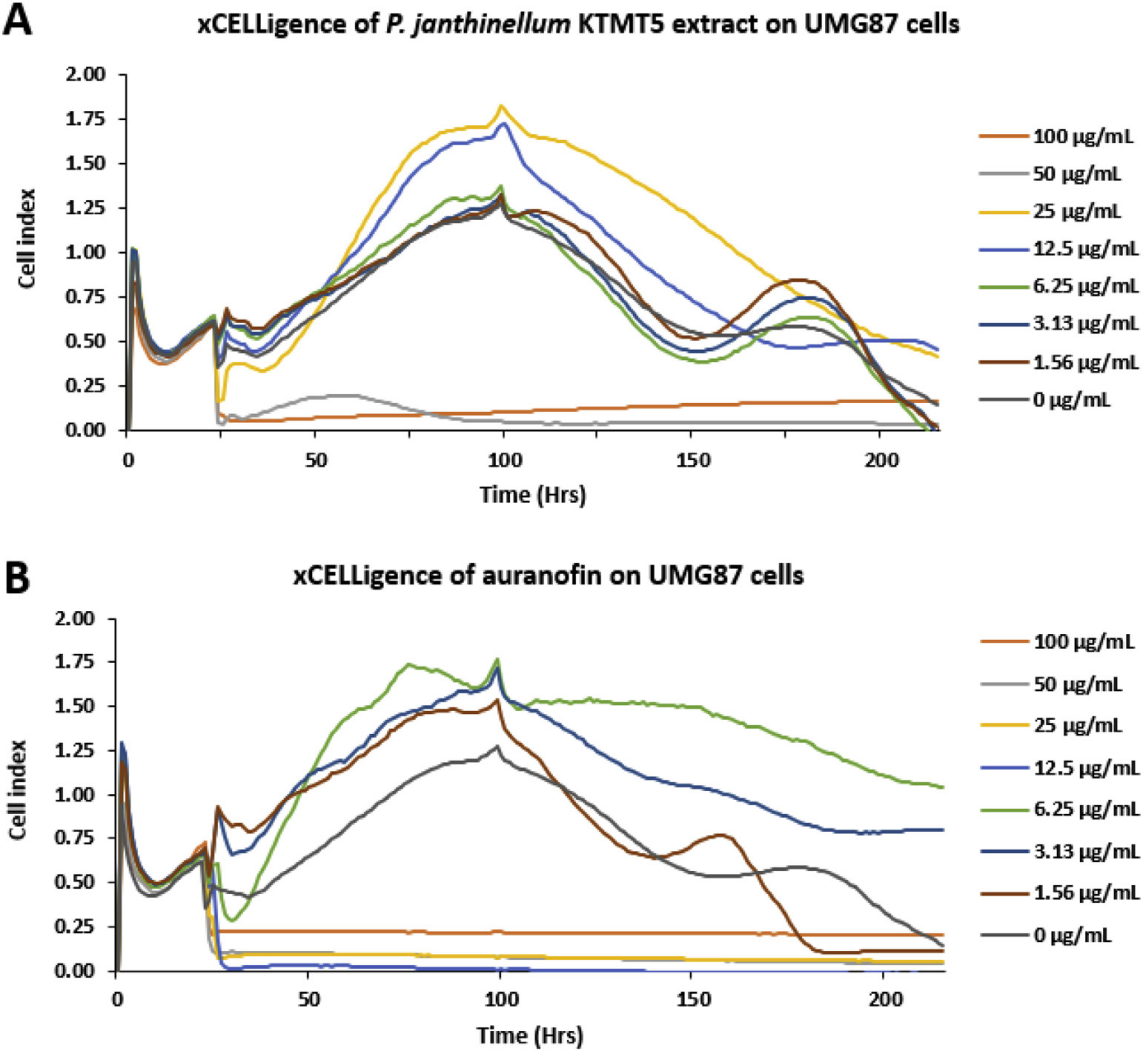
Real time cell analyzer (RTCA) xCELLigence assay of the crude extract of *P. janthinellum* KTMT5 extract on UMG87 cells is shown in **A** above. The cells were monitored for 250 hours. Auranofin was the positive control as shown in **B** while the untreated cells (0 μg/mL) served as the negative control in both **A** and **B**.

Fig. 3 shows the effect of the crude extract of *P. janthinellum* KTMT5 on UMG87 cells compared with that of auranofin on the same cell line in the real time cell analyzer (RCTA) xCELLigence assay. In Fig. 3 (A), the profile shows that after the treatment of UMG87 cells with the fungal extract at the 24th hour of incubation, there was a decline in cell viability as shown by cell index readings of approximately 0.06 for both the 50 and 100 μg/mL treatments. The UMG87 cells in the 100 μg/mL fungal extract treatment began to recover just before the 50th hour as shown by the slight increase in cell index up to 0.14 at the termination of the experiment on the 250th hour of incubation. The cells in the 50 μg/mL fungal extract treatment maintained a steady profile from the 100th hour and recorded a cell index of 0.05 at the termination of the experiment. Fig. 3 (B) shows the anticancer profile of auranofin which was used as a positive control in the experiment. The raw data used in plotting Fig. 3 can be found in Supplementary File 3.

**Fig. 4:**
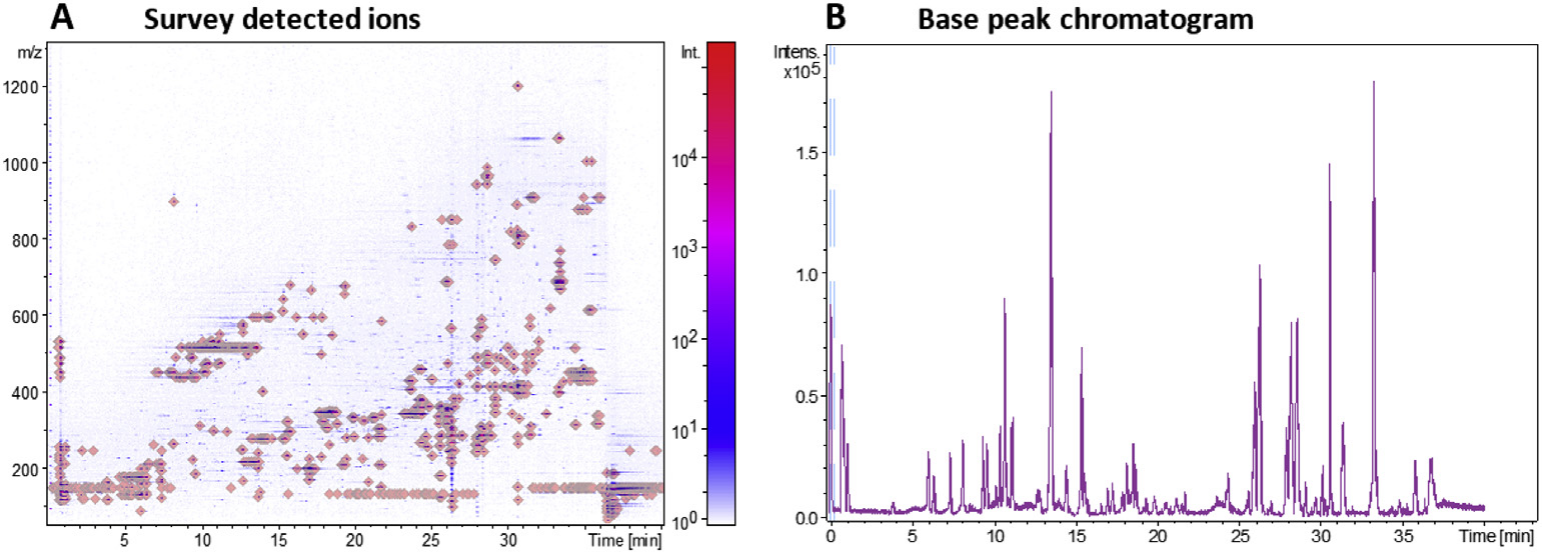
The survey view of ions detected by LC-QOF-MS/MS (**A**), and the base peak chromatogram (**B**) of the crude extract *P. janthinellum* KTMT5.

The secondary metabolite compounds in the crude extract of *P. janthinellum* KTMT5 were analysed in an untargeted approach using LC-QTOF-MS/MS. Fig. 4 shows the survey view of the detected in an analytic run of 40 minutes and the resulting base peak chromatogram.

A list of putatively identified metabolites from metabolite profiling of the crude extract of *P. janthinellum* KTMT5 is shown in Table 2. Supplementary File 4 contains raw spectral data generated using the LC-QTOF-MS/MS system.

**Table 2:** Secondary metabolite compounds of *P. janthinellum* KTMT5.

RT (min)	Meas. *m/z*	Calc. *m/z*	Error ppm	Adducts	Compound	Formulae	Biological source
0.72	512.18	512.181	1	[M + CH_3_OH + H]^+^	Penicilloic acid	C_16_H_20_N_2_O_5_S_1_	*P. chrysogenum* [3]
0.75	486.1675	486.1669	1	[M+Na]^+^	Pivampicillin	C_22_H_29_N_3_O_6_S_1_	*?*
0.76	153.0558	153.0552	4	[M + H–H_2_O]^+^	Penicillic acid	C_8_H_10_O_4_	*P. chrysogenum* [3]
0.80	153.0068	153.0061	4	[M+K]^+^	Muscimol	C_4_H_6_N_2_O_2_	*Amanita muscaria* [4]
16.97	167.0697	167.0695	1	[M+2H + Na]^3+^	Aurovertin D	C_25_H_32_O_9_	*Metarhizium anisopliae* [5]
17.04	221.0685	221.0687	−1	[M+K]^+^	*N*-Formylloline	C_9_H_14_N_2_O_2_	*Acremonium coenophialum* [6]
17.16	661.0842	661.0826	2	[M + H–2H_2_O]^+^	Verticillin A	C_30_H_28_N_6_O_6_S_4_	*Clonostachys rosea* [7]
17.80	493.182	493.1809	2	[M+2Na–H]^+^	Terretonin F	C_26_H_30_O_8_	*Aspergillus insuetus* [8]
26.13	263.1841	263.1853	4	[M + CH_3_OH + H]^+^	Talaromycin A	C_12_H_22_O_4_	*T. stipitatus* [9]
30.08	407.2184	407.219	1	[M + NH_4_]^+^	Roquefortine C	C_22_H_23_N_5_O_2_	*P. chrysogenum* [10]

**Fig. 5:**
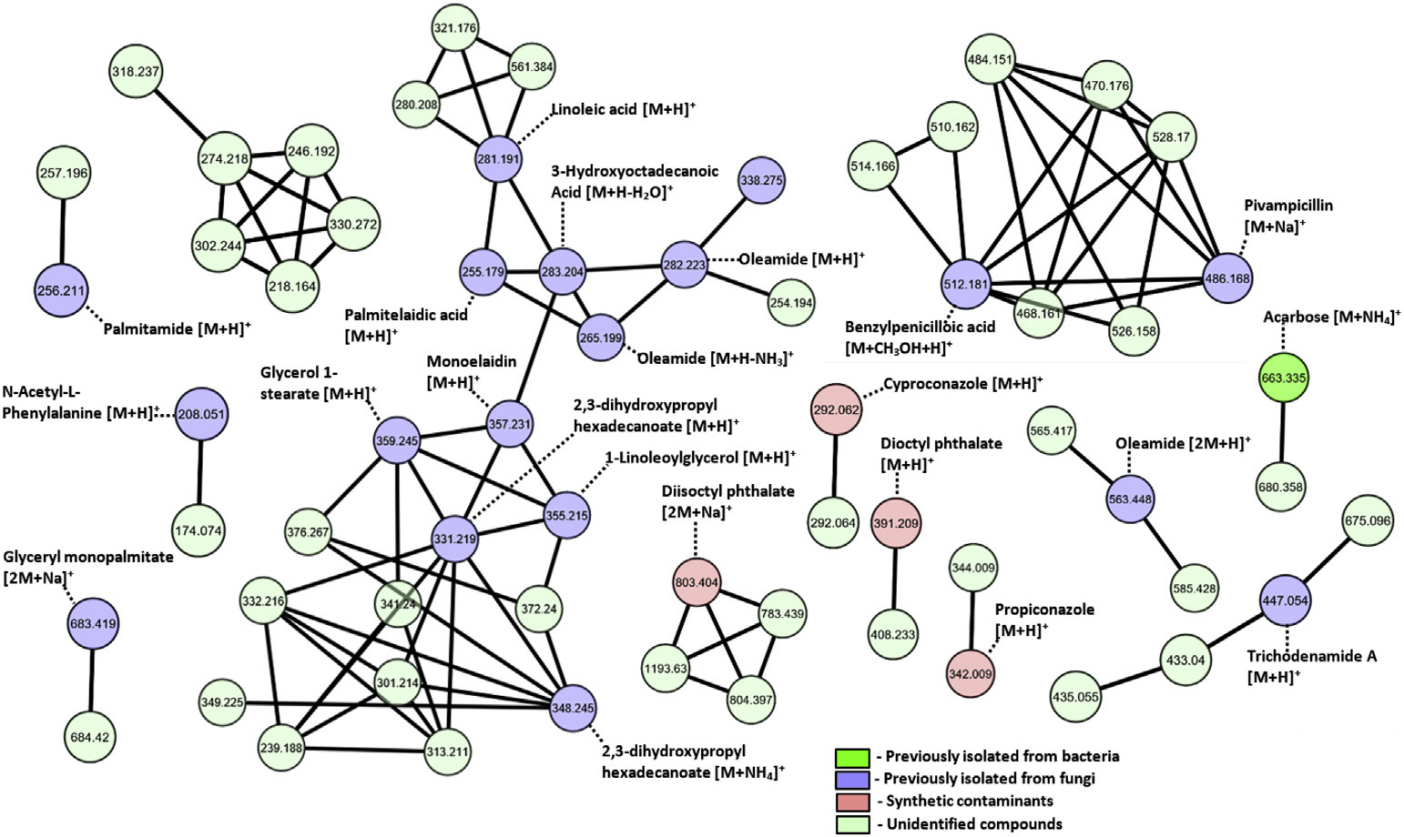
Molecular network of metabolites in the crude extract of *P. janthinellum* KTMT5.

Fig. 5 shows the molecular network of metabolites in the crude extract of *P. janthinellum* KTMT5. Molecular networking resulted in 3810 detected hits, 904 identified hits and 45 unique hits. Acarbose, a compound produced by bacteria of the genus Actinoplanes was detected [1]. Synthetic contaminants which include propiconazole, diisobutyl phthalate and dioctyl phthalate commonly occur from plasticware used during experiments [2]. Supplementary Files 5 and 6 contain data and parameters used to generate the molecular network.

## 2 Experimental design, materials, and methods

### 2.1 Collection of mine tailing material, isolation and identification of fungi

Mine tailings were collected from an inactive tailings heap in Springs, Johannesburg, South Africa (26°13′7.08″S, 28°29′8.64″E). A disinfected auger was used to drill 30 cm deep into the mine tailings heap where about 100 g of the tailings were collected. Samples were transported to the laboratory in sterile sealable plastic bags and were processed within 24 hours of collection. Isolation of fungi was done by weighing 10 g of the mine tailings into 100 mL of sterile phosphate buffered saline (PBS) that was then mixed by vortexing. This was then serially diluted to 10^−9^ and plated on potato dextrose agar. Culture plates were incubated at 25 °C and for 14 days. DNA extraction was then performed using the ZR Fungal/Bacterial DNA Kit™ (Zymo Research, Irvine, CA, USA), following the manufacturer's instructions, followed by polymerase chain reaction (PCR) amplification of the ITS region of ribosomal DNA (rDNA) using the ITS1 (5´-TCCGTAGGTGAACCTGCGG-3´) and ITS4 (5´-TCCTCCGCTTATTGATATGC-3´) primer pair. Forward and reverse direction sequencing was performed using the ABI PRISM™ 3500xl Genetic Analyzer (Thermo Fisher Scientific, Inc., Waltham, MA, USA). Purification of sequencing products was done using ZR-96 DNA Sequencing Clean-up Kit™ (Zymo Research, Irvine, CA, USA). ITS1 and ITS4 sequences of each isolated fungi were then individually analysed to identify the isolated fungi by searching for closely related fungi on GenBank database using the Nucleotide Basic Local Alignment Search (BLASTN) search tool.

### 2.2 Fermentation and extraction of crude secondary metabolite compounds

The fungus *P. janthinellum* KTMT5 was grown in 3 L of potato dextrose broth (Potato infusion 200 g/L, dextrose 20 g/L) for 21 days at 28 °C in an orbital shaking incubator (Amerex Gyromax, Temecula, CA, USA) at 150 rpm. Secondary metabolites were extracted from the broth by firstly filtering out the mycelium using a double layered muslin cloth and mixing the filtrate with an equal volume of ethyl acetate. The organic solvent phase that formed after allowing the mixture to stand for an hour was collected and concentrated under reduced pressure at 40 °C. The concentrated crude extracts were then stored at – 20 °C.

### 2.3 MTS assay on A549 and UMG87 cells

The MTS [3-(4,5-dimethylthiazol-2-yl)-5-(3-carboxymethoxyphenyl)-2-(4-sulfophenyl)-2H-tetrazolium] cytotoxicity assay was performed on A549 (lung carcinoma) and UMG87 (glioblastoma cells) in 96 well plates with Dulbecco's modified eagle medium (Gibco, Carlsbad, CA, USA) in 15% heat inactivated fetal bovine serum (Merck, Johannesburg, SA) [11].

Initially, the wells were seeded with at 5 × 10^4^ cells/mL and incubated for 24 hours at 37 °C in 5% CO_2_ (v/v), then the crude fungal extract of *P. janthinellum* KTMT5 prepared in dimethyl sulfoxide (DMSO) was then introduced at increasing logarithmic concentrations which were as follows: 3.13, 6.25, 12.5, 25, 50 and 100 μg/mL. The treated cells were then further incubated for 96 hours under the same conditions, during which no media or fungal extract was further added. Auranofin was included in the experiment as a positive control and was tested in the same way as the fungal extract. Untreated cells (0 μg/mL) were included to serve as negative controls. At the end of the incubation period, 5 μl of MTS (Promega, Madison, WI, USA) was added to each well and the absorbance of the MTS formazan product was measured at 490 nm after 1-, 2- and 4-h incubation periods [12]. Cell viability was then calculated using the formulae below where E_a_ is absorbance of the extract, B_a_ is absorbance of the blank and C_a_ is the absorbance of the negative control:

% Cell Viability = [(E_a_ - B_a_)/(C_a_ - B_a_)] × 100

The IC_50_ values were then calculated in GraphPad Prism software (v. 7.05, GraphPad Software, Inc., La Jolla, CA, USA) using non-linear regression analysis of cell viability data.

### 2.4 xCELLigence real-time cell analyzer (RTCA) assay on U87MG cells

The xCELLigence RTCA assay was performed on 96 well electronic plates precoated with gold microelectrodes (E-Plate® 96, ACEA Biosciences Inc., San Diego, CA, USA). The UMG87 cells were initially seeded with 1 × 10^5^ cells/mL and allowed to grow for 24 hours at 37 °C in 5% CO_2_ (v/v). The crude extract of *P. janthinellum* KTMT5 and the positive control (auranofin) were dissolved in DMSO and then introduced at increasing logarithmic concentrations as described earlier: 3.13, 6.25, 12.5, 25, 50 and 100 μg/mL. Untreated cells (0 μg/mL) were included to serve as negative controls. The cells were monitored for up to 250 hours without supplementation of media or fungal extract during the incubation period. Impedance measurements taken every 15 minutes to monitor cell viability. The data was retrieved, and a graphic representation of the bioactivity was reproduced.

### 2.5 Metabolite profiling using LC-QTOF-MS/MS and molecular networking

Untargeted metabolite profiling of the crude extract of *P. janthinellum* KTMT5 was done using a liquid chromatography coupled to quadrupole time-of-flight with tandem mass spectrometry (LC-QTOF-MS/MS) system in positive mode [13]. This system has an ultra-high-performance liquid chromatography (Dionex UltiMate 3000, Thermo Scientific, Germany) connected to a QTOF (Compact™, Bruker Daltonics, Germany) with an electrospray ionization (ESI) interface. The fungal extract was prepared for analysis by dissolving it in HPLC grade methanol at 1 mg/mL (w/v) and filtering through 0.22 μm polyvinylidene fluoride (PVDF) membrane syringe filter. A set volume of 5 μL was injected into the system and chromatographic separation of analytes in reverse phase was achieved using a Raptor ARC-18 column with dimensions of 2.7 μm (particle size), 2.1 mm (internal diameter), 100 mm (length) and 90 Å (pore size) (Restek, Bellefonte, PA, USA). The mobile phase consisted of solvent A [0.1% formic acid in H_2_O (v/v)] and solvent B [0.1% formic acid in acetonitrile (v/v)]. The flow of the mobile phase was set up as shown in Table 3 and the QTOF system parameters are shown in Table 4.

**Table 3:** Mobile phase flow settings of reverse phase HPLC.

Step	Retention time (min)	Solvent	
Constant flow	0 → 2	95%	Solvent A
		5%	Solvent B
Gradient flow	2 → 30	95 → 5%	Solvent A
		5 → 95%	Solvent B
Gradient flow	30 → 31	5 → 95%	Solvent A
		95 → 5%	Solvent B
Constant flow	31 → 40	95%	Solvent A
		5%	Solvent B

**Table 4:** Parameters of the QTOF-MS/MS system.

Feature	Acquisition Parameter
Source type	Electrospray ionization
Ion polarity	Positive
Scan	50–1300 *m/z*
Set capillary	4500 V
Set end plate offset	- 500 V
Set charging voltage	2000 V
Set nebulizer	1.8 Bar
Set dry heater	220 °C
Set dry gas	2.5 L/min
Set APCI heater	0 °C

Spectral data was acquired and processed using Compass DataAnalysis software version 4.3 (Bruker Daltonics Germany). MetFrag 2.1 was used in the putative identification of metabolites by linking to three databases, namely PubChem, 2 ChemSpider^3^ and KEGG Compound^4^ [13]. The MetFrag settings used were as follows: Database search relative mass deviation (Search ppm) = 10.0; precursor ion = [M+H]^+^; fragment peak match absolute mass deviation (Mzabs) = 0.01; fragment peak match relative mass deviation (Mzppm) = 10; charge = positive and mode = [M+H]^+^. Molecular networking using an online workflow at the global natural products social molecular networking platform [14]. The parameters used to generate the network are summarized in Supplementary File 1.

## Footnotes

1 https://msbi.ipb-halle.de/MetFragBeta/.

2 https://pubchem.ncbi.nlm.nih.gov/.

3 http://www.chemspider.com/.

4 https://www.genome.jp/kegg/compound/.
