# Supplementary material for: Anticancer activity and metabolite profiling data of Penicillium janthinellum KTMT5
Source: Data Brief. 2019 Dec 7;28:104959. doi: 10.1016/j.dib.2019.104959 (PMC6928313; doi:10.1016/j.dib.2019.104959)
Supplement: Multimedia component 1 [file mmc1.docx]

>**MH660411.1 *Arcopilus aureus* isolate KTMT1** internal transcribed spacer 1, partial sequence; 5.8S ribosomal RNA gene, complete sequence; and internal transcribed spacer 2, partial sequence

CCGCTCCCCTGGAAAGCCCCTGTGGCCGCCCGGGGCTGCGAGCCCCCCGGCCCCCCTCGCGGGGGCGCCGCCGGAGGATACCCAACTCTTGATTATTTTAGGCCTCTCTGAGTCTTCTGTACTGAATAAGTCAAAACTTTCAACAACGGATCTCTTGGTTCTGGCATCGATGAAGAACGCAGCGAAATGCGATAAGTAATGTGAATTGCAGAATTCAGTGAATCATCGAATCTTTGAACGCACATTGCGCCCGCCAGTATTCTGGCGGGCATGCCTGTTCGAGCGTCATTTCAACCATCAAGCCCCAGGCTTGTGTTGGGGACCTGCGGCTGCCGCAGGCCCTGAAATCCAGTGGCGGGTTCGCTGTCACCCCGAGCGTAGTAGCAATATCTCGCTCAGGGCGTGCTGCGGGCACCGGCCGTTAAAAGCTGCCTTCTGGCAACACCCAA

>**MH660412.1 *Penicillium janthinellum* isolate KTMT2** internal transcribed spacer 1, partial sequence; 5.8S ribosomal RNA gene, complete sequence; and internal transcribed spacer 2, partial sequence

GGAAGGATCATTACCGAGTGAGGGCCCTCTGGGTCCAACCTCCCACCCGTGTTTATCATACCTAGTTGCT

TCGGCGGGCCCGCCGTCAGGCCGCCGGGGGGCATCCGCCCCCGGGCCCGCGCCCGCCGAAGCCCCCCCTGAACGCTGTCTGAAGATTGCAGTCTGAGCGATTAGCTAAATCAGTTAAAACTTTCAACAACGGATCTCTTGGTTCCGGCATCGATGAAGAACGCAGCGAAATGCGATAAGTAATGTGAATTGCAGAATTCAGTGAATCATCGAGTCTTTGAACGCACATTGCGCCCCCTGGTATTCCGGGGGGCATGCCTGTCCGAGCGTCATTGCTGCCCTCAAGCACGGCTTGTGTGTTGGGCCCCCGCCCCCCGGCTCCCGGGGGGCGGGCCCGAAAGGCAGCGGCGGCACCGCGTCCGGTCCTCGAGCGTATGGGGCTTCGTCACCCGCTCTGTAGGCCCGGCCGGCGCCCGCCGGCGACCCCCCTC

>**MH660413.1 *Penicillium oxalicum* isolate KTMT4** small subunit ribosomal RNA gene, partial sequence; internal transcribed spacer 1 and 5.8S ribosomal RNA gene, complete sequence; and internal transcribed spacer 2, partial sequence

TTCCGTAGGGTGAACCTGCGGAAGGATCATTACCGAGTGAGGGCCCTCTGGGTCCAACCTCCCACCCGTGTTTATCGTACCTTGTTGCTTCGGTGAGSCCGCCTCACGGCCGCCGGGGGGCATCTGCCCCCGGGCCCGCGCTCGCCGAAGACACACAAACGAACTCTTGTCTGAAGATTGCAGTCTGAGTACTTGACTAAATCAGTTAAAACTTTCAACAACGGATCTCTTGGTTCCGGCATCGATGAAGAACGCAGCGAAATGCGATAAGTAATGTGAATTGCAGAATTCAGTGAATCATCGAGTCTTTGAACGCACATTGCGCCCCCTGGTATTCCGGGGGGCATGCCTGTCCGAGCGTCATTGCTGCCCTCAAGCACGGCTTGTGTGTTGGGCTCTCGCCCCCCCGTCTCGGGAGGGCGGGCCCGAAAGGCAGCGGCGGCACCGCGTCCGGTCCTCGAGCGTATGGGGCATCGTCACCCGCTCTGTAGGCCCGGCCGGCGCCCGCCGGCGAACACCAATCAATCTTAACCA

>**MH660414.1 *Penicillium janthinellum* isolate KTMT5** small subunit ribosomal RNA gene, partial sequence; internal transcribed spacer 1 and 5.8S ribosomal RNA gene, complete sequence; and internal transcribed spacer 2, partial sequence

GTAGGTGAACCTGCGGAAGGATCATTACCGAGTGAGGGCCCTCTGGGTCCAACCTCCCACCCGTGTTTATCATACCTAGTTGCTTCGGCGGGCCCGCCGTTAGGCCGCCGGGGGGCACCCGCCCCCGGGCCCGCGCCCGCCGAAGCCCCCCCTGAACGCTGTCTGAAGATTGCAGTCTGAGCGATTAGCTAAATCAGTCAAAACTTTCAACAACGGATCTCTTGGTTCCGGCATCGATGAAGAACGCAGCGAAATGCGATAAGTAATGTGAATTGCAGAATTCAGTGAATCATCGAGTCTTTGAACGCACATTGCGCCCCCTGGTATTCCGGGGGGCATGCCTGTCCGAGCGTCATTKCTGCCCTCAAGCACGGCTTGTGKGTTGGGCCCCCGCCCCCCGGCTCCCGGGGGGCGGGCCCGAAAGGCAGCGGCGGCACCGCGGKCCGGTCCTCGAGCGTATGGGGCTTCGT

>**MH660415.1 *Acidiella americana* isolate KTMT6** small subunit ribosomal RNA gene, partial sequence; internal transcribed spacer 1 and 5.8S ribosomal RNA gene, complete sequence; and internal transcribed spacer 2, partial sequence

TCCGTAGGTGAACCTGCGGAGGGATCATTACCGAGTGGAGGGCCTCCGGGCCCGACCTCCAACCCTTGTGTTATCCGACCTCTGTTGCCTCGGGGGCGGCCCGGCCCTCGCGCCGGGGCCCCCCGCGGACCCCTCTGGAAACTCTTGCATCTCTGCGTCTGAGTATGATTTTGAATCAATCAAAACTTTCAACAACGGATCTCTTGGTTCCAGCATCGATGAAGAACGCAGCGAAATGCGATAAGTAATGTGAATTGCAGAATTCAGTGAATCATCGAATCTTTGAACGCACATTGCGCCCCTCGGCATTCCGGGGGGCATGCCTGTTCGAGCGTCATTAACACCACTCAAGCCTCGTCTTGGTATTGGGCGTCCCGCGGGGCCACACCCGCGCGCCTCCAAATCTCCGGSTGAGCCGTCCGTCTCTAAGCGTTGTGGA
